# Long-Gap Esophageal Atresia Gross Type C and D: A Retrospective Study of Surgical Management and Postoperative Complications Within the First Year of Life in the Nordic Countries

**DOI:** 10.3390/children12030363

**Published:** 2025-03-14

**Authors:** Ann Christine Waarkjær Olsen, Antti Koivusalo, Ragnhild Emblem, Audun Mikkelsen, Jan F. Svensson, Anna Maria Tollne, Markus Almström, Linus Jönsson, Helene Lilja, Felipe Donoso, Thorstein Sæter, Jørgen Mogens Thorup, Pernilla Stenström, Einar Arnbjörnsson, Niels Qvist

**Affiliations:** 1 Research Unit for Surgery, and Centre of Excellence in Gastrointestinal Diseases and Malformations in Infancy and Childhood (GAIN), Odense University Hospital, 5000 Odense, Denmark; ann.christine.w.olsen@rsyd.dk; 2 Faculty of Health Sciences, Clinical Institute, University of Southern Denmark, 5230 Odense, Denmark; 3 Department of Pediatric Surgery, Children’s Hospital, University of Helsinki, 00100 Helsinki, Finland; antti.koivusalo@hus.fi; 4 Institute of Clinical Medicine, University of Oslo, 0313 Oslo, Norway; remblem@ous-hf.no; 5 Section for Pediatric Surgery, Oslo University Hospital, 5000 Oslo, Norway; 6 Department of Pediatric Surgery, Karolinska University Hospital, 17177 Stockholm, Sweden; Jan.f.svensson@regionstockholm.se (J.F.S.); annamaria.tollne@regionstockholm.se (A.M.T.); markus.almstrom@ki.se (M.A.); 7 Department of Women’s and Children’s Health, Karolinska Institute, 17177 Stockholm, Sweden; 8 Department of Pediatric Surgery, Queen Silvia Children’s Hospital, 41650 Gothenburg, Sweden; linus.jonsson@vgregion.se; 9 Department of Pediatric Surgery, Children’s Hospital, Uppsala University, 75185 Uppsala, Sweden; helene.lilja@kbh.uu.se (H.L.); felipe.donoso@kbh.uu.se (F.D.); 10 Department of Women’s and Children’s Health, Uppsala University, 75310 Uppsala, Sweden; 11 Department of Pediatric Surgery, St. Olavs Hospital, Trondheim University Hospital, 7030 Trondheim, Norway; thorstein.seter@stolav.no; 12 Department of Pediatric Surgery, Rigshospitalet, Copenhagen University Hospital, 2100 Copenhagen, Denmark; joergen.mogens.thorup@regionh.dk; 13 Department of Pediatrics, Children’s Hospital, Lund University, 22100 Lund, Swedeneinar.arnbjornsson@telia.com (E.A.); 14 OPEN, Odense Patient Data Explorative Network, Odense University Hospital, 5000 Odense, Denmark

**Keywords:** esophageal atresia, long-gap, Gross type C, Gross type D, surgical management—postoperative complication

## Abstract

Objective: Several surgical approaches are being used in the reconstruction of long-gap esophageal atresia. We investigated which methods are being used in the Nordic countries and the postoperative complications that occurred in the first year of life. Methods: This study is a retrospective multicenter study, where medical records on children with esophageal atresia Gross type C or D born in the period from 1 January 2000 to 1 May 2017 were reviewed. Results: Forty-four patients were included in this study, forty-three with Gross type C and one with Gross type D. Thirty-six patients were included in the statistical analysis. Delayed esophageal anastomosis was performed in half of the patients and an esophageal replacement procedure in the other half. Postoperative complications were common, but there was no difference in postoperative complications or weight-gain within the first year of life. There were no differences in hospital stay or duration of parenteral nutrition. Attempted primary esophageal anastomosis was significantly more common in patients that underwent an esophageal replacement procedure compared to those who underwent a delayed esophageal anastomosis. Conclusions: No significant relationship between surgical approach and postoperative complications within the first year of life could be demonstrated. Long-term functional studies are warranted.

## 1. Introduction

Esophageal atresia encompasses a spectrum of five different types (A–E) according to the Gross classification and occurs in between 1.27 and 4.55 per 10,000 births [1,2]. In more than 85% of the cases, esophageal atresia is combined with a distal trachea-esophageal fistula (TEF) in Gross type C and D, and where type D is very rare. Traditionally, long-gap esophageal atresia is defined by the absence of intra-abdominal air on a plain X-ray typical for Gross type A and B or a gap length between the upper and lower pouches corresponding to the height of three vertebras or more irrespective of the type of atresia [3]. The inability to achieve sufficient length on the two pouches to perform a primary anastomosis may be another definition and, in a recent publication, this was shown to occur in 5% of the cases with Gross type C atresia [4]. The condition is clinically challenging, and one must choose between delayed esophageal anastomosis or esophageal replacement procedures. The replacement procedure may include a gastric pull-up or an interposition procedure with the jejunum, colon, or a gastric tube [5,6,7]. There is no high grade of evidence on which of the methods is preferred [8] and the choice of procedure will often be based upon the discretion by the surgeon or the institution. Most of the publications on long-gap esophageal atresia are on Gross Type A without a distal tracheo-esophageal fistula, and available results from cases with a fistula are more scarce and often difficult to differentiate from the overall results on long-gap esophageal atresia. A systematic review from the APSA Outcomes and Evidence-Based Practice Committee concluded that the 18 provided recommendations were based on level 4–5 evidence [3].

The aim of the present study was to review the surgical procedures used in children with a long-gap esophageal atresia in Gross type C or D in the Nordic countries (Norway, Sweden, Finland, Denmark) over a 17-year period and to compare the characteristics of postoperative complications in the first year of life associated with the two different surgical approaches.

## 2. Materials and Methods

### 2.1. Study Population

Medical records on children with esophageal atresia (ICD-10 code DQ39) and born in the period from 1 January 2000 to 1 May 2017 were retrieved from each patient registry at each of the participating hospitals and reviewed to identify patients with Gross type C or D atresia, where it was not possible to perform a primary esophageal anastomosis.

### 2.2. Study Design

The records were searched for gestational age (GA), mode of delivery, birthweight and -length, and associated congenital anomalies. The exposure variable was the surgical approach with delayed esophageal anastomosis with or without an active elongation technique, gastric pull-up (GPU), formation of a gastric tube, or interposition procedure (colonic, ileal, or jejunal interposition).

### 2.3. Outcomes

Outcome variables were postoperative complications with anastomotic stricture, anastomotic leakage, gastroesophageal reflux (GER), and mortality. GER was defined as symptomatic reflux and/or endoscopically verified inflammation. Anastomotic stricture was defined as a narrowing of the esophagus causing dysphagia and requiring dilatation. Finally, we registered the number of days admitted at the hospital, number of thoracotomies, and days on parenteral nutrition within the first year of life together with children’s gain in weight and length at one year of age.

### 2.4. Setting

Data were collected from 9 pediatric surgical departments in Norway, Sweden, Finland, and Denmark. Each department had 1–3 persons responsible for the data collection, who were also authorized to enter the collected data into the database. The data were entered via an encrypted connection and stored securely by using the online system Research Electronic Data Capture (RedCap) provided by the research infrastructure Odense Patient data Exploration Network (OPEN).

### 2.5. Statistical Analysis

Data were summarized and are displayed as descriptive statistics. Results on categorical variables are presented as frequencies and percentages, while results on numeric variables are presented as means and standard deviations (SDs). To test whether a result on continuous variables was significant, we used Student‘s *t*-test, and for categorical, binary outcome variables, we used Fisher’s exact test. Due the relatively small number of patients, the four types of esophageal interposition techniques and gastric pull-up were pooled into one group and compared to the group of patients where a delayed esophageal anastomosis was performed. Missing values were excluded from the analyses of outcome variables. A *p*-value <0.05 was considered statistically significant. Stata 17.0 was used for the analysis.

## 3. Results

We included forty-four patients with Gross type C (43 patients) and D (one patient). Eleven patients were from Norway, fifteen from Sweden, five from Finland, and thirteen from Denmark (Table 1).

Of the forty-four patients, eight were excluded from the statistical analysis, because they died before the final reconstruction procedure. Four patients died of sepsis after a thoracotomy with attempted primary anastomosis and with closure of the tracheal fistula and four from other comorbidities. Four of these patients were from Denmark, three were from Norway, and one was from Finland (Table 2). Half of the 36 patients included in this study underwent a delayed esophageal anastomosis and the other half a replacement procedure. Information on the initial gap length was available in 61% of the patients in both groups. The gap length was, on average, 2.7 cm in the group with delayed esophageal anastomosis and 3.8 cm in the replacement group. This difference was not statistically significant.

We found no significant differences in gestational age, method of delivery, or associated congenital anomalies between the two treatment groups. Birth weight and length were significantly lower in those who underwent delayed esophageal anastomosis compared to the esophageal replacement procedure with *p*-values of 0.019 and 0.003, respectively.

All patients were managed by open surgery. A closure of the distal fistula and the placement of a gastrostomy tube was performed at the primary therapy except for a few cases with an early primary replacement. An attempted primary anastomosis at thoracotomy was significantly more often performed in patients who later underwent an esophageal interposition procedure (*p* = 0.007). The mean age at reconstruction was 151 days for the delayed esophageal anastomosis group and 250 days in the interposition group. This difference was not significant.

The most common esophageal interposition procedure was GPU (66.7%). The most used approach in Norway was a delayed esophageal anastomosis while the most used approach in Finland and Denmark was an interposition procedure. In Sweden, the use of both methods was almost equal (Table 3). Only three patients (6.8%) received active elongation technique treatment prior to the final reconstruction. One had a Kimura procedure while the other two were managed with external traction sutures.

The frequency of postoperative complications was relatively high in both treatment groups (Table 4). The most common postoperative complication for the delayed esophageal anastomosis group was anastomotic stricture in 83.3% of the patients, while the most common complication for the esophageal replacement procedure was GER, which was reported in 72.2% of the patients. There was no difference in postoperative mortality between the two groups, with two deaths occurring after delayed esophageal anastomosis (11.1%) and two deaths (11.1%) after the esophageal replacement procedure.

## 4. Discussion

This study showed that delayed esophageal anastomosis and esophageal interposition procedures including gastric pull-up were used equally in the Nordic countries but with some differences among the countries. In Norway, patients were most often managed by delayed esophageal anastomosis, whereas interposition procedures were most common in Denmark and Finland. In Sweden, the distribution between the two methods was almost equal. In a previous study from the same observation period and from the same hospitals including Gross type A and B with long-gap esophageal atresia, we found a higher frequency of the interposition method in Finland compared to other countries [9]. In a recent study on long-gap esophageal Gross type C, two-thirds underwent a delayed esophageal and one-third a replacement procedure [4].

The birth weight and length were significantly lower in patients receiving delayed primary esophageal anastomosis, which may indicate that low birthweight might be a proxy for choosing delayed primary anastomosis. The surgical approach and method for reconstruction were primarily based upon the discretion by the surgeon and the institutional preference and experience, which also explains the variety of treatments between the institutions and countries. The patient records contained no specific explanation of the chosen surgical procedure and there were no specific criteria or information on the gap-length for the choice of surgical method. Although a delayed primary anastomosis is generally recommended in long-gap esophageal atresia [10], the patients with a tracheoesophageal fistula constitutes a special problem because the patients often undergo surgery in the neonatal period with an attempt at primary reconstruction, where an approximation of the two esophageal pouches is impossible for various reasons and therefore calls for alternative reconstruction methods. An interesting finding in our study was that an attempt at primary esophageal anastomosis was significantly more often associated with a replacement procedure. Damage to the native esophagus could be an explanation. Another interesting finding was that there were no significant differences in the duration of parenteral nutrition or weight and length between the two groups at the 1-year follow-up. It was not possible to collect detailed information on the indications for parenteral nutrition.

Postoperative complications were common in both treatment groups but seems to be similar to other studies on long-gap esophageal atresia, although results vary considerably. In our study, anastomotic leakage occurred in 27.8%, which is comparable to the 28.76% found in a systematic review with delayed esophageal anastomosis for long-gap esophageal atresia [11] and where half of the patients had a distal tracheo-esophageal fistula. In primary esophageal anastomosis with no long-gap, a frequency of 19.4% has been reported [12]. For the replacement procedure, the frequency is best documented for gastric tube replacement with a frequency as low as 10% [13]. The risk of anastomotic leakage should in theory be lower in the replacement group due to less tension on the anastomosis, but our results did not reveal any obvious underlying condition as a risk factor for anastomotic stricture. The rate of leakage incidents may be dependent upon the use of contrast studies and some incidents may be subclinical. In the present study, only clinical leakages with saliva or milk in the plural drain or a perianastomotic collection that needed drainage was included. A leakage often heals spontaneously, but 2 out of the 10 patients with anastomotic leakage underwent a surgical revision, and both patients were in the interposition group only.

The most frequent postoperative complication in our study was anastomotic stricture, occurring in 69.4% of the patients, and this is comparable to the 57% reported in a review including studies with delayed esophageal anastomosis [11] but higher than the 23.2% reported for esophageal repair in general [14]. Differences in the definition of stricture are a major problem in the reporting of anastomotic stricture. In our study, the definition was a narrowing requiring one or more dilatation. The presence of GER is a known risk factor for the development of anastomotic stricture, and this was found in 55.6% of cases in our study with a tendency for a higher frequency in the group with a replacement procedure.

In a recent systematic review comparing gastric transposition or esophageal lengthening with delayed esophageal anastomosis in the repair of a long-gap esophageal atresia, a tendency for a lower complication rate with the delayed esophageal anastomosis was found [15].

All the patients in the present study underwent open surgery. With the introduction of the thoracoscopic repair of esophageal atresia, a different approach to obtain delayed primary esophageal anastomosis may be developed where the sutured elongation may advance a delayed primary esophageal anastomosis [16]. A future perspective in the reconstruction of long-gap esophageal atresia in patients with a tracheoesophageal fistula will depend on whether the primary surgical approach is open or thoracoscopic. With the open approach, the fistula should be closed before waiting for the natural growth/elongation of the pouches. If one goes for an attempt of primary reconstruction, a gastric pull-up seems to be the most appropriate alternative. With the thoracoscopic approach, the fistula should be closed with or without suture traction. Under all circumstances, the child should undergo sham-feeding as this may promote growth of the upper pouch and the maturation of the swallowing reflex [17]. After sufficient growth of the pouches and narrowing of the gap, an alternative to the conventional sutured anastomosis may be a magnetic anastomosis [18]. In cases where salvage of the native esophagus is not possible, a gastric conduit may be the recommended approach due to its simplicity and long-term durability [19]. International collaborative studies are necessary to gain more evidence for this treatment algorithm.

### Limitations

The present study has a retrospective design and a long study period of 17 years where treatment may have changed over time. We may also have failed to identify a few potential patients due to code errors in the patient registries. Surgeon experience and clinical assessment in the chosen surgical procedure were not registered. The amount of data for each event were insufficient for a reliable analysis of normality. In those patients who underwent an elongation procedure, the information on the number of elongation procedures or the duration of elongation in each patient was not obtained from the records. Another limitation was the pooling of the different replacement procedures into one group.

## 5. Conclusions

Delayed esophageal anastomosis and esophageal replacement procedures were being used equally in the Nordic countries. The most common esophageal replacement procedure used was the gastric pull-up. An attempt at primary esophageal anastomosis was significantly more often associated with a replacement procedure. No significant difference in postoperative complications between the two groups within the first year of life was found.

## Figures and Tables

**Table 1:** The number of patients registered in the different departments and born between 1st of January 2000 and 1st of May 2017.

Department	Country	Gross Type C (*n* = 43)	Gross Type D (*n* = 1)
Oslo University Hospital, Rikshospitalet and Ullevål	Norway	7	0
St. Olavs Hospital, Trondheim University Hospital	Norway	4	0
Children’s Hospital, Uppsala	Sweden	1	0
The Queen Silvia Children’s Hospital, Sahlgrenska University Hospital, Gothenburg	Sweden	4	0
Karolinska University Hospital Solna, Stockholm	Sweden	8	0
Children’s Hospital, Lund	Sweden	2	0
Children’s Hospital, Helsinki University	Finland	4	1
Copenhagen University Hospital	Denmark	0	0
Odense University Hospital	Denmark	13	0

**Table 2:** Patient characteristics in the two treatment groups.

Patient Characteristics	Total N = 36	Delayed Esophageal Anastomosis N = 18	Esophageal Replacement Procedure N = 18	*p*-Value
Gestational age (mean ± SD)	33.2 ± 4.0	32.3 ± 5.2	34.2 ± 1.9	0.153 *
Birth weight (g) (mean ± SD)	1933.8 ± 785.7	1632.9 ± 856.9	2234.7 ± 587.1	0.019 *
Birth length (cm) (mean ± SD)	43.0 ± 5.7	39.9 ± 6.5	45.8 ± 3.0	0.003 *
Mode of delivery (%)				
Caesarian section	40%	50%	29%	0.305 **
Vaginal	60%	50%	71%	
Other congenital anomalies, *n* (%)				
Chromosomal abnormality	2 (5.6%)	0	2 (11.1%)	0.486 **
Congenital heart defect ^a^	12 (33.3%)	7 (38.9%)	5 (27.8%)	0.725 **
Other anomalies ^b^	26 (72.2%)	10 (55.5%)	16 (88.9%)	0.06 **
Attempted primary anastomosis, *n* (%)	17 (47.2%)	4 (22.2%)	13 (72.2%)	0.007 **
Age at final reconstruction (days) (mean ± SD)	200.4 ± 204.8	151.1 ± 159.6	249.8 ± 236.1	0.151 *

^a^ Requiring medical or surgical treatment. ^b^ Other anomalies were mainly anal and duodenal atresia, kidney anomalies, pulmonal anomalies, ventricular septum defects, atrial septum defects, and vertebral anomalies. SD = standard deviation. * Student’s *t*-test. ** Fisher’s exact test.

**Table 3:** Surgical methods used in the different countries.

Surgical Method	Total (*n* = 36)	Norway (*n* = 8)	Sweden (*n* = 15)	Finland (*n* = 4)	Denmark (*n* = 9)
Delayed primary anastomosis *n* (%)	18 (50%)	7 (87.5%)	8 (53.3%)	1 (25%)	2 (22.2%)
Esophageal replacement procedure *n* (%)	18 (50%)	1 (12.5%)	7 (46.7%)	3 (75%)	7 (77.8%)
Gastric pull-up, *n* (%)	12 (66.7%)	0	4 (57.1%)	1 (33.3%)	7 (100%)
Gastric tube, *n* (%)	4 (22.2%)	0	3 (42.9%)	1 (33.3%)	0
Colonic interposition, *n* (%)	1 (5.6%)	1 (100%)	0	0	0
Jejunal interposition, *n* (%)	1 (5.6%)	0	0	1 (33.3%)	0
Death before final reconstruction *n* (%)	8 (18.2%)	3 (27.3%)	0	1 (20%)	4 (30.8%)

**Table 4:** Postoperative complications and 1-year follow up data after delayed primary anastomosis and the esophageal replacement procedure.

Postoperative Complications and 1-Year Follow-Up Data	Total (*n* = 36)	Delayed Primary Anastomosis (*n* = 18)	Esophageal Replacement Procedure (*n* = 18)	*p*-Value
Anastomotic leakage	10 (27.8%)	6 (33.3%)	4 (22.2%)	0.711 **
Re-operation ^a^	2 (20%)	0	2 (100%)	0.133 **
Anastomotic stricture	25 (69.4%)	15 (83.3%)	10 (55.6%)	0.146 **
Number of dilatation procedures ^b^ (mean ± SD)	9.6 ± 8.8	11.1 ± 10.2	7.4 ± 6.0	0.318 *
Gastroesophageal reflux	20 (55.6%)	7 (38.9%)	13 (72.2%)	0.092 **
Nissen fundoplication ^c^	5 (25%)	2 (40%)	3 (60%)	1.000 **
Other complications ^d^	16 (44.4%)	6 (33.3%)	10 (55.6%)	0.315 **
Total number of thoracotomies (mean ± SD)	2.3 ± 0.9	2.4 ± 0.8	2.2 ± 1.1	0.480 **
Days of hospital admission (mean ± SD)	184.9 ± 104.7	183.8 ± 98.9	186.1 ± 113.2	0.950 *
Duration of parenteral nutrition (days) (mean ± SD)	47.9 ± 55.8	56.3 ± 68.4	41.1 ± 43.9	0.459 *
Weight gained at 1-year follow-up (g) (mean ± SD)	5789.8 ± 1350.9	6218.3 ± 1357.0	5295.4 ± 1209.6	0.071
Length gained at 1-year follow-up (cm) (mean ± SD)	26.8 ± 7.6	30.4 ± 7.5	24.2 ± 6.8	0.095 *
Mortality, *n* (%)	4 (11.1%)	2 (11.1%)	2 (11.1%)	1.000 **

^a^ Reoperation because of anastomotic leakage. ^b^ Dilatations because of anastomotic stricture. ^c^ Nissen fundoplication because of GER. ^d^ Other complications were mainly pneumothorax, pneumonia, sepsis, and chylothorax. SD, standard deviation. * Student’s *t*-test. ** Fisher’s exact test.

## Data Availability

The original contributions presented in this study are included in the article. Further inquiries can be directed at the corresponding author.
